# Effect of Graphene Doping Level near the Metal Contact Region on Electrical and Photoresponse Characteristics of Graphene Photodetector

**DOI:** 10.3390/s20174661

**Published:** 2020-08-19

**Authors:** Jaedong Jung, Honghwi Park, Heungsup Won, Muhan Choi, Chang-Ju Lee, Hongsik Park

**Affiliations:** School of Electronics Engineering, Kyungpook National University, Daegu 41566, Korea; showmmee99@knu.ac.kr (J.J.); hoepark@ee.knu.ac.kr (H.P.); soby617@knu.ac.kr (H.W.); mhchoi@ee.knu.ac.kr (M.C.)

**Keywords:** graphene, graphene-metal contact, photodetector, built-in electric field, junction barrier height, doping level difference

## Abstract

Graphene-metal contact is crucial to fabricate high-performance graphene photodetectors since the external quantum efficiency (EQE) of the photodetector depends on the contact properties, and the influence of the contact properties is particularly dominant in short channel devices for high-speed applications. Moreover, junction properties between the channel graphene and graphene near the contact are also important to analyze the photoresponse because the built-in electric field in the junction determines the EQE of the photodetector. In this study, we investigated a relation between the photoresponse and the built-in electric field induced from the doping level difference in the junction between the channel graphene and graphene near the contact. The photoresponse could be enhanced with a high junction barrier height that is tuned by the doping level difference. In addition, we observed that the improved electrical characteristics of channel graphene do not guarantee the enhancement of the photoresponse characteristics of graphene photodetectors.

## 1. Introduction

Graphene, a two-dimensional honeycomb lattice of carbon atoms, has attracted attention due to its unique electrical and optical characteristics [1,2]. The unique characteristics stem from the zero-bandgap structure of graphene, which enables ultra-broadband optical applications from the ultraviolet to microwave regime [3]. In addition, the supreme carrier transport properties of graphene enable an ultrafast operation of electronic and optical devices [4,5]. Moreover, the compatibility of graphene with mature complementary metal-oxide-semiconductor platforms has received considerable interest in that graphene-based low-cost/high-performance devices can be easily integrated on a single chip. In accordance with such properties, graphene-based photodetectors [6,7,8,9,10,11], optical modulators [12,13], and electronic devices [14,15] have been reported. In particular, graphene has been actively used for photodetectors due to its high-speed operation [7,9], and broad optical bandwidth [8]. However, the responsivity of graphene photodetectors is limited to tens of mA/W because of the weak optical absorption of single-layer carbon atoms [11]. To improve the responsivity of graphene photodetectors, therefore, various approaches have been suggested, including absorption-assisted lead sulfide quantum dots [16], graphene-semiconductor hybrid structures [17,18,19,20], and photoconductive nanostructures [10]. Recently, it has been reported that the generation of a significant photocurrent was observed at graphene-metal contacts or graphene p-n junctions, resulting from the band bending [21,22,23,24,25,26]. Since the effect of the contact or junction on the photocurrent is more dominant with the channel length scaled down, further study is required to understand the photoresponse characteristics of graphene photodetectors. 

In this work, we investigated the effect of the doping level difference between the channel graphene and graphene near the metal contact on the photoresponse of a graphene photodetector. We observed that the electrical characteristics and doping levels of channel graphene were changed by the post-annealing process, and we investigated the relation between the photoresponse and the electrical characteristics of channel graphene at various annealing temperatures. Finally, we separately analyzed the photoresponse characteristics of the channel and contact region of the graphene photodetector.

## 2. Experimental Methods

**Figure 1:**
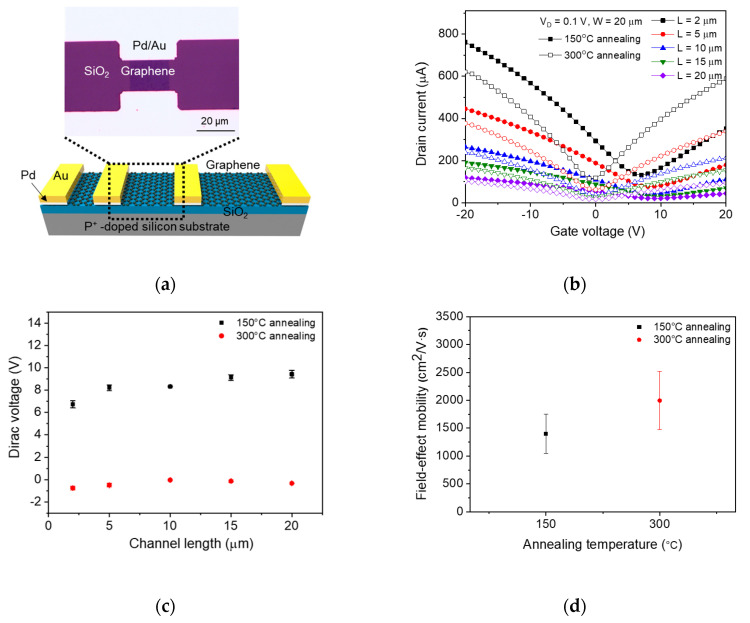
(**a**) Schematic and optical image of the field-effect transistor (FET)-structure graphene photodetector. (**b**) Two sets of transfer characteristics (I_D_–V_G_) of the graphene photodetectors measured at V_D_ = 0.1 V after 150 °C and 300 °C annealing. Channel lengths varied from 2–20 μm, and channel width was fixed to 20 μm. The error bar indicates a standard deviation. (**c**) Dirac voltage vs. channel length of the graphene photodetectors extracted from the I_D_–V_G_ characteristics measured after 150 °C and 300 °C annealing. (**d**) Field-effect mobility of the graphene photodetectors after the post-annealing process. The mobility values were calculated using the maximum transconductance values and conventional field-effect mobility equation [27].

We fabricated graphene photodetectors with various channel lengths using commercially available monolayer graphene (Graphene Platform, Tokyo, Japan). Graphene was transferred on the thermally oxidized silicon substrate. The thickness of the SiO_2_ layer was 100 nm. Photolithography was used for patterning the electrode on the graphene layer. A palladium (Pd)/gold (Au) (20 nm/50 nm) was deposited on the patterned photoresist by an e-beam evaporating system and followed by the lift-off process. Then, we defined the channel region of the graphene photodetectors by using photolithography and oxygen plasma treatment (30 W, 60 mTorr, 180 sec). The photoresist was removed by acetone and isopropanol. A top-view optical image of the fabricated graphene photodetector with a channel length of 15 μm and a channel width of 20 μm is shown in the top of Figure 1a.

Prior to the electrical measurement, we annealed the fabricated graphene photodetectors in a vacuum probe station (~10^−7^ Torr) for two hours. The electrical and photoresponse characteristics of the graphene photodetectors were measured using a semiconductor parameter analyzer (Agilent 4155A, Santa Clara, CA, USA) and a 1550-nm-wavelength infrared laser diode (Thorlabs LPSC-1550-FC, Newton, NJ, USA). The laser diode was connected to the laser diode controller (Thorlabs LDC205C, Newton, NJ, USA) and temperature controller (Thorlabs TED200C, Newton, NJ, USA) for stable operation.

## 3. Results and Discussion

We firstly measured the electrical characteristics of the graphene photodetectors with various channel lengths (2–20 μm) after a 150 °C-annealing process. The graphene photodetectors were annealed again at 300 °C, and we remeasured the electrical characteristics after the first measurement. A schematic image of the graphene photodetector with various channel lengths is shown in the bottom of Figure 1a. This annealing process has been widely used to remove surface impurities (PMMA residue, photoresist residue and moisture), which act as p-type dopants [28,29,30]. These impurities induce unwanted doping on graphene, thereby resulting in a positive shift of Dirac voltage [28]. The 150 °C-annealing process is commonly used to evaporate moisture from graphene, and the 300 °C-annealing process is known to effectively remove polymer residue on graphene [29,30]. Figure 1b shows two sets of transfer characteristics (I_D_–V_G_) of the graphene photodetectors measured at a constant drain-source bias (V_D_ = 0.1 V). The gate bias was varied from −20 V to 20 V. The channel lengths of the graphene photodetectors were 2, 5, 10, 15, and 20 μm and the channel width was fixed at 20 μm. The graphene photodetectors annealed at 150°C had a positively shifted Dirac voltage (~ 8 V), whereas the graphene photodetectors reannealed at 300 °C had a Dirac voltage close to 0 V. This indicated that the 150 °C annealing evaporated moisture and the 300 °C annealing effectively removed the polymer residue, which is critical for the practical applications with a low operating voltage of graphene photodetectors. One experimental detail for the graphene FETs is hysteresis. We evaluated the hysteresis of the graphene photodetectors after 300 °C annealing. The hysteresis of the 10-μm- and 20-μm-channel photodetectors were –1.2 V and –1.1 V, respectively. More details for hysteresis characteristics of the graphene photodetectors are shown in Supplementary Material. The Dirac voltages of the graphene photodetectors with various channel lengths are shown in Figure 1c. When the devices were annealed at 150 °C, an increasing trend of the Dirac voltage with channel length was significantly observed, while a negligible channel-length dependence was shown after 300 °C annealing. This channel-length dependence of the Dirac voltage was induced by the doping level difference between the channel graphene and the graphene near the metal contact (we denoted the graphene near the metal contact as “contact graphene”). The Dirac voltage of the graphene annealed at 150 °C positively increased with channel length since the influence of p-type dopants became stronger by increasing the portion of remaining polymer residue on the channel graphene. After 300 °C annealing, the channel-length dependence was relatively reduced. The weak channel-length dependence was observed because the contact graphene was still n-type doped by the absorption and bonding with the metal contact of Pd [31]. Therefore, the Dirac voltage of graphene photodetectors after 300 °C annealing was negatively shifted. The increase in the absolute values of the Dirac voltage for the relatively short-channel graphene photodetectors also indicated that the contact graphene was n-type doped. In addition, we compared the field-effect mobility at various annealing temperatures because mobility is generally used to evaluate the electrical properties of graphene. As shown in Figure 1b, the graphene photodetectors annealed at 300 °C exhibited a sharper transfer curve than those annealed at 150 °C, which indicates that the 300 °C-annealed graphene photodetectors have higher field-effect mobility than 150 °C-annealed photodetectors. Figure 1d shows the field-effect mobility of the graphene photodetectors depending on the annealing temperature. The field-effect mobility was calculated by using the maximum transconductance value, and the equation for calculating the field-effect mobility is represented by

(1) $$\mu_{FE}=\frac{g_{m}L_{ch}}{C_{ox}WV_{D}},$$

where, *g_m_* is the transconductance, *L_ch_* is the channel length, *C_ox_* is the gate oxide capacitance, *W* is the channel width, and *V_D_* is the drain bias [27]. The graphene photodetectors annealed at 150 °C had an average field-effect mobility of ~1400 cm^2^/V∙s, while the graphene photodetectors reannealed at 300 °C showed an improved value of ~2000 cm^2^/V∙s. Thus, this annealing process for removing undesirable doping is essential for important material parameters that determine the electrical properties of graphene. 

**Figure 2:**
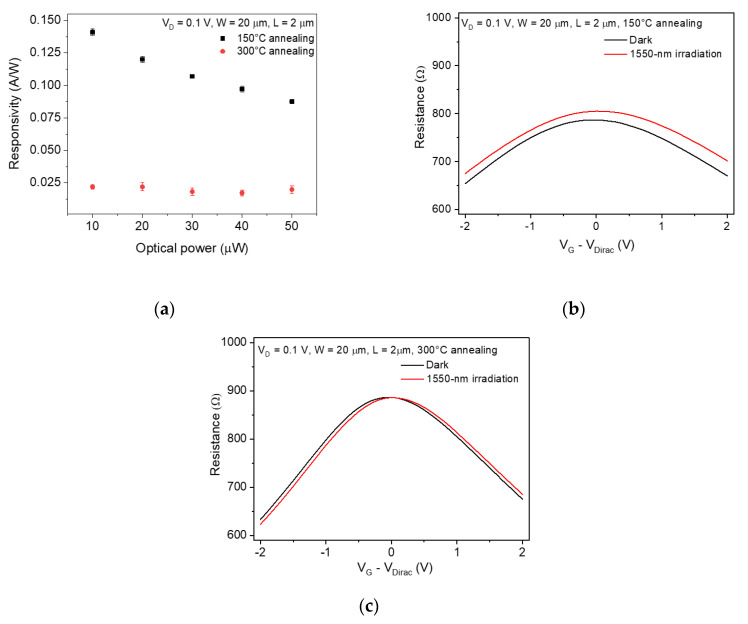
(**a**) Responsivity of the graphene photodetectors with the channel length of 2 μm as a function of optical power after annealing at 150 °C and 300 °C. The error bar indicates a standard deviation. Total device resistance of the graphene photodetector as a function of gate bias measured after (**b**) 150 °C and (**c**) 300 °C annealing under dark and illuminated condition.

We expected that the improved electrical properties of the graphene photodetector would induce enhanced photoresponse characteristics. However, the photoresponse characteristics were degraded after 300 °C annealing rather than 150 °C annealing. Figure 2a shows the photoresponsivity of the graphene photodetector with the 2 μm channel length after 150 °C and 300 °C annealing under various irradiation conditions. Under the light illumination with an optical power of 10 μW, the photoresponsivity of the 150 °C-annealed graphene photodetector was 0.141 A/W at the gate bias of 8 V, while it decreased to 0.022 A/W at the gate bias of −2 V after the 300 °C annealing.

To find the reason that the photoresponsivity was degraded after the 300 °C annealing even though the electrical properties of graphene had improved, we investigated the optical modulation of the total device resistance of the graphene photodetector at different annealing temperatures. Figure 2b,c show the total device resistance of the 2 μm channel graphene photodetectors as a function of gate bias after the 150 °C and 300 °C annealing, respectively. The optical power was 50 μW. The graphene photodetector that was annealed at 150 °C had a change ratio in the total resistance of approximately 5% at V_G_ – V_Dirac_ of 2 V, whereas that of the 300 °C-annealed photodetector was near 0%. These results show that the 300 °C-annealing process affects the quantum efficiency of the graphene photodetector, thus resulting in degradation of the photoresponsivity. The photoresponse can generally occur in the channel region and near the graphene-metal contact region under light illumination [26]. 

**Figure 3:**
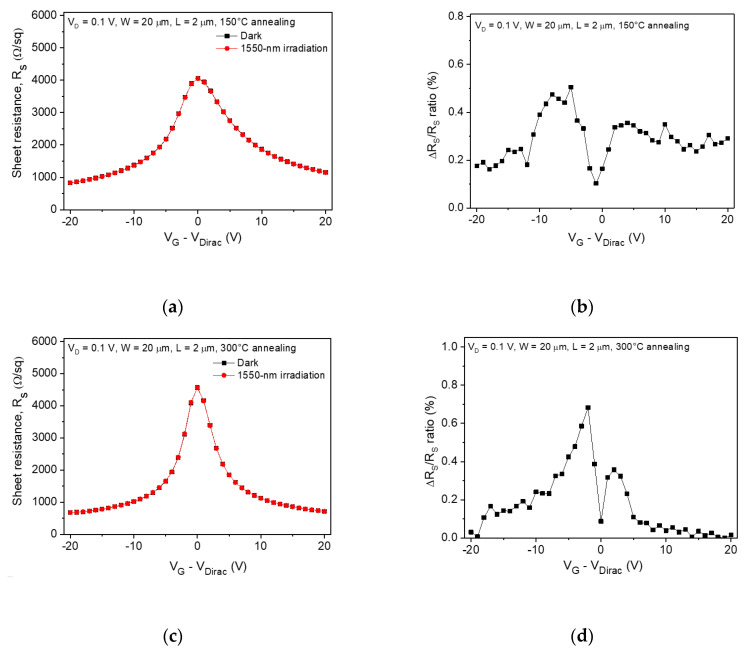
Sheet resistance as a function of gate bias extracted from the total device resistance measured after (**a**) 150 °C and (**c**) 300 °C annealing under dark and illuminated conditions. (**b**), (**d**) Change ratio in the sheet resistance as a function of gate bias calculated from (**a**) and (**c**), respectively.

In order to compare the photoresponse characteristics of the channel and contact regions, we extracted the sheet and contact resistance from the total device resistance using the transmission line method (TLM), which has been typically used to evaluate the graphene-metal contact resistance [21]. Figure 3a shows the sheet resistance of the 150 °C-annealed graphene photodetector under dark and light illumination, where the negligible photoresponse was observed in the gate bias range from –20 to 20 V. A calculated change ratio in the sheet resistance as a function of the gate bias is shown in Figure 3b, where the maximum value of the change ratio was under 1%. Figure 3c shows the sheet resistance of graphene as a function of the gate bias after 300 °C-annealing process. There were no significant changes except that the slope of the sheet resistance curve was sharper than after the 150 °C annealing, which meant that the carrier mobility in channel graphene increased, as shown in Figure 1d. The optical modulation of sheet resistance was also negligible under light illumination, and the change ratio in sheet resistance was under 1% (Figure 3d). These results showed that the channel region had a very small contribution to the photoresponse of the graphene photodetector.

**Figure 4:**
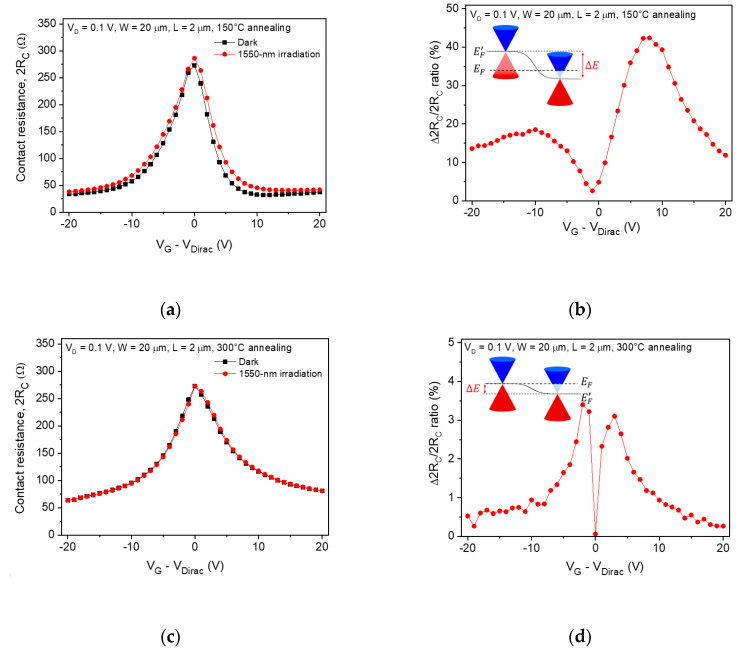
Contact resistance as a function of gate bias extracted from the total device resistance measured after (**a**) 150 °C and (**c**) 300 °C annealing under dark and illuminated conditions. (**b**), (**d**) The change ratio in the contact resistance as a function of gate bias calculated from (**a**) and (**c**), respectively. Inset: band diagrams in the junction between the channel graphene (left) and graphene near the metal contact (right). The Fermi energy level (E_F_) and graphene Dirac point energy level (E^’^_F_) are illustrated by the dashed and solid lines, respectively.

In contrast to the sheet resistance, a noticeable optical modulation of the contact resistance was observed under light illumination. Figure 4a shows the contact resistance of the graphene photodetector as a function of gate bias after 150 °C annealing under dark and light illumination. Unlike the sheet resistance of graphene, the contact resistance was significantly modulated by the light illumination. This relatively large modulation was attributed to the increase in contact resistance, which was very similar to the optical modulation of the total device resistance. The result showed that the photoresponse of the graphene photodetector was dominantly affected by the optical modulation of the contact graphene region. The increase in the contact resistance was attributed to the carrier-carrier scattering due to excess photogenerated carriers, thus resulting in a decrease in the carrier mobility [10]. From the calculation result, the field-effect mobility of the graphene photodetector with the 2 μm channel length decreased from 950 (under dark) to 890 cm^2^/V∙s (under illumination). Figure 4b shows the change ratio in contact resistance as a function of gate bias. The change ratio shows an asymmetric shape for electrons and holes. This asymmetric conduction behavior was caused by excess resistance in the graphene p-n junction [24]. The maximum value of the change ratio in contact resistance was 42% at V_G_ – V_Dirac_ = 7 V. Under light illumination, the photogenerated electrons and holes can be separated according to the induced electric field as determined by the doping level difference between the channel and contact graphene (inset of Figure 4b). After 150 °C annealing, the channel graphene layer was p-type doped with a carrier concentration of 1.73 × 10^12^ cm^−2^, and the Fermi level of the channel graphene was located in the valance band (0.17 eV far from Dirac point). Because the Fermi level of the contact graphene is located in the conduction band, this large doping level difference can induce a large built-in electric field. However, the contact resistance of the graphene photodetector was not significantly modulated by light illumination after 300 °C annealing, as shown in Figure 4c. The change ratio in contact resistance drastically decreased to 3.5% (Figure 4d), which was attributed to the lowering of junction barrier height between the channel and contact graphene.

From the electrical measurements (Figure 1b,c), we estimated that the channel graphene was slightly n-type doped after 300 °C annealing. The junction barrier height between the channel and contact graphene was relatively lower than that of the graphene photodetector after 150 °C annealing because the doping level difference was very small, as shown in the inset of Figure 4d. Photogenerated carriers recombine right away due to the short lifetime of graphene [32,33]. As we expected, the field-effect mobility of graphene was not significantly changed from 1170 (under dark) to 1160 cm^2^/V∙s (under light illumination) after 300 °C annealing.

## 4. Conclusions

We have investigated the effect of the doping level difference between the channel graphene and graphene near the metal contact on the photoresponse of graphene photodetectors. The photoresponse was found to be related to the junction barrier height because this determines the magnitude of the electric field in the junction. We observed that the post-annealing process for improving the electrical characteristics of channel graphene adversely affects the photoresponse of the graphene photodetector, although the annealing process increased the carrier mobility of graphene and removed the unwanted doping effects. Thus, to fabricate a high-responsivity graphene photodetector, the height of the junction barrier between the channel graphene and contact graphene must be properly designed, considering the graphene doping level in each region.

## Supplementary Materials

The following are available online at https://www.mdpi.com/1424-8220/20/17/4661/s1, Figure S1: the hysteresis characteristics of the 300 °C-annealed graphene FETs with the channel lengths of 10 μm and 20 μm.
