# Supplementary material for: Effect of Graphene Doping Level near the Metal Contact Region on Electrical and Photoresponse Characteristics of Graphene Photodetector
Source: Sensors (Basel). 2020 Aug 19;20(17):4661. doi: 10.3390/s20174661 (PMC7506932; doi:10.3390/s20174661)
Supplement: Supplementary file 1 [file sensors-20-04661-s001.pdf]

Supplementary Material

Effect of graphene doping level near the metal contact region on electrical and photoresponse characteristics of graphene photodetector

Jaedong Jung, Honghwi Park, Heungsup Won, Muhan Choi, Chang-Ju Lee * and Hongsik Park *

School of Electronics Engineering, Kyungpook National University, Daegu 41566, Korea; showmmee99@knu.ac.kr (J.J.); hoepark@ee.knu.ac.kr (H.P.); soby617@knu.ac.kr (H.W.); mhchoi@ee.knu.ac.kr (M.C.);

*Correspondence: chjlee@knu.ac.kr (C.-J.L.); hpark@ee.knu.ac.kr (H.P.); Tel.: +82-53-940-8819 (C.-J.L.); +82-53-950-5519 (H.P.)

The Figure S1 shows the hysteresis characteristics of the 300°C-annealed graphene FETs with the channel lengths of 10 μm and 20 μm. The gate voltage sweep started at –20 V. The hysteresis of the 10-μm- and 20-μm-channel FETs are –1.2 V and –1.1 V, respectively.

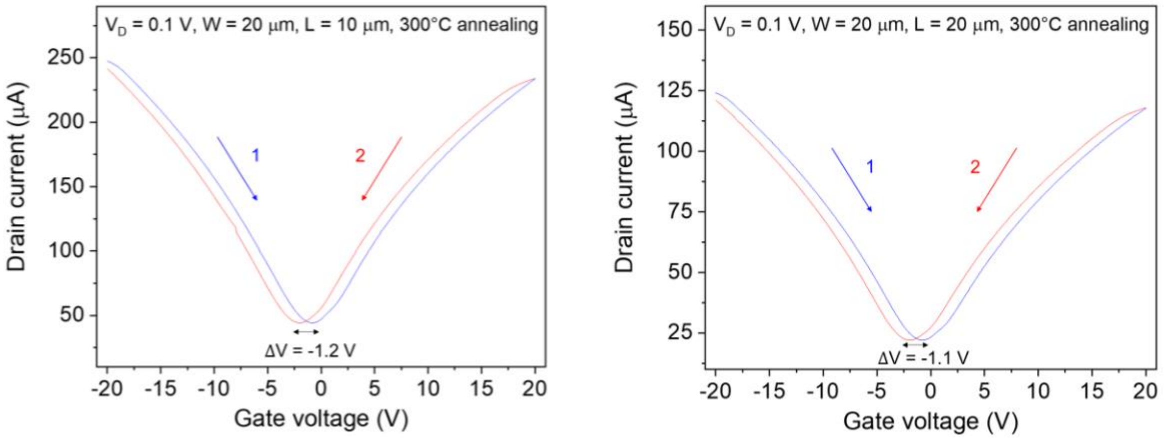

(**a**) (**b**)

Figure S1. The hysteresis characteristics of the 300°C-annealed graphene FETs with the channel lengths of (a) 10 μm and (b) 20 μm. The gate voltage was swept continuously from –20 V to 20 V and back to –20 V with a sweep rate of 10 V/s.

The total amount of effective charge trapping can be simply estimated by the amount of Dirac voltage shift [1]. The hysteresis of –1.2 V and –1.1 V were equivalent to the trapped charge density of 2.59×10^{11} cm^{-2} and 2.37×10^{11} cm^{-2}, respectively. These hysteresis significantly reduced than typical graphene-FETs built on a graphene/SiO_2/Si of 17 V and have similar trap density with the Si/SiO_2 interface in conventional silicon transistors [1,2]. This implies that the 300°C annealing process could effectively suppress the hysteresis of the graphene FETs. However, the annealing process could not remove the hysteresis completely as shown in Figure S1. In order to realize the hysteresis free devices, the high-k material or two-dimensional insulator has been attracted as gate dielectrics because they could enhance the gate controllability and charge trapping speed [3,4].

References

1. Lee, Y.G.; Kang, C.G.; Jung, U.J.; Kim, J.J.; Hwang, H.J.; Chung, H.J.; Seo, S.; Choi, R.; Lee, B.H. Fast transient charging at the graphene/ SiO_2 interface causing hysteretic device characteristics. *Appl. Phys. Lett.* **2011**, *98*, 5–7, doi:10.1063/1.3588033.

2. Wang, H.; Wu, Y.; Cong, C.; Shang, J.; Yu, T. Hysteresis of electronic transport in graphene transistors. *ACS Nano* **2010**, *4*, 7221–7228, doi:10.1021/nn101950n.

3. Cadore, A.R.; Mania, E.; Watanabe, K.; Taniguchi, T.; Lacerda, R.G.; Campos, L.C. Thermally activated hysteresis in high quality graphene/*h*-BN devices. *Appl. Phys. Lett.* **2016**, *108*, doi:10.1063/1.4953162.

4. Carrion, E.A.; Serov, A.Y.; Islam, S.; Behnam, A.; Malik, A.; Xiong, F.; Bianchi, M.; Sordan, R.; Pop, E. Hysteresis-free nanosecond pulsed electrical characterization of top-gated graphene transistors. *IEEE Trans. Electron Devices* **2014**, *61*, 1583–1589, doi:10.1109/TED.2014.2309651.
